# High-flow nasal cannula to prevent postextubation respiratory failure in high-risk non-hypercapnic patients: a randomized multicenter trial

**DOI:** 10.1186/s13613-017-0270-9

**Published:** 2017-05-02

**Authors:** Rafael Fernandez, Carles Subira, Fernando Frutos-Vivar, Gemma Rialp, Cesar Laborda, Joan Ramon Masclans, Amanda Lesmes, Luna Panadero, Gonzalo Hernandez

**Affiliations:** 1 Critical Care Department, Hospital Sant Joan de Deu- Fundacio Althaia, CIBERES, Universitat Internacional de Catalunya, Dr Joan Soler 1, 08243 Manresa, Spain; 2 CIBERES, Hospital de Getafe, Madrid, Spain; 3 Hospital Son Llatzer, Majorca, Spain; 4 Hospital Valle Hebron, Barcelona, Spain; 5 Hospital del Mar, CIBERES, IMIM, Pompeu Fabra University, Barcelona, Spain; 6 Hospital Virgen de la Salud, Toledo, Spain

**Keywords:** Mechanical ventilation, Weaning, Reintubation, High-flow oxygen

## Abstract

**Background:**

Extubation failure is associated with increased morbidity and mortality, but cannot be safely predicted or avoided. High-flow nasal cannula (HFNC) prevents postextubation respiratory failure in low-risk patients.

**Objective:**

To demonstrate that HFNC reduces postextubation respiratory failure in high-risk non-hypercapnic patients compared with conventional oxygen.

**Methods:**

Randomized, controlled multicenter trial in patients who passed a spontaneous breathing trial. We enrolled patients meeting criteria for high-risk of failure to randomly receive HFNC or conventional oxygen for 24 h after extubation. Primary outcome was respiratory failure within 72-h postextubation. Secondary outcomes were reintubation, intensive care unit (ICU) and hospital lengths of stay, and mortality. Statistical analysis included multiple logistic regression models.

**Results:**

The study was stopped due to low recruitment after 155 patients were enrolled (78 received high-flow and 77 received conventional oxygen). Groups were similar at enrollment, and all patients tolerated 24-h HFNC. Postextubation respiratory failure developed in 16 (20%) HFNC patients and in 21 (27%) conventional patients [OR 0.69 (0.31–1.54), *p* = 0.2]. Reintubation was needed in 9 (11%) HFNC patients and in 12 (16%) conventional patients [OR 0.71 (0.25–1.95), *p* = 0.5]. No difference was found in ICU or hospital length of stay, or mortality. Logistic regression models suggested HFNC [OR 0.43 (0.18–0.99), *p* = 0.04] and cancer [OR 2.87 (1.04–7.91), *p* = 0.04] may be independently associated with postextubation respiratory failure.

**Conclusion:**

Our study is inconclusive as to a potential benefit of HFNC over conventional oxygen to prevent occurrence of respiratory failure in non-hypercapnic patients at high risk for extubation failure.

Registered at Clinicaltrials.gov NCT01820507.

**Electronic supplementary material:**

The online version of this article (doi:10.1186/s13613-017-0270-9) contains supplementary material, which is available to authorized users.

## Background

The need for mechanical ventilation (MV) is one of the main reasons for admission to intensive care units (ICU). Once patients recover from critical illness, they need to be extubated and resume spontaneous breathing. It is difficult to predict whether a patient is ready to be extubated [1, 2], and physicians must balance the benefits of prolonging MV allowing for better recovery, against the associated risks, mainly pulmonary infections, delirium, and muscle atrophy. Between 10 and 20% of attempts to extubate fail [3], and extubation failure is associated with increased morbidity and mortality [4]. Thus, there is a need for strategies that can reduce the rate of extubation failure [5].

After extubation, patients routinely receive oxygen-enriched air through nasal prongs or masks; the concentration of oxygen is controlled and progressively tapered off within hours or days based on patients’ tolerance.

Recently, a new method to deliver oxygen, high-flow nasal cannula (HFNC), reached the clinical arena [6, 7]. HFNC devices supply between 30 and 60 L/min of a controlled mixture of actively warmed (32–37 °C) and humidified (up to 100% relative humidity) oxygen and air through modified nasal prongs, producing a moderate positive end-expiratory pressure (PEEP) [8]. HFNC might help prevent extubation failure through different mechanisms. First, the controlled oxygen concentration may reduce transient hypoxemic episodes [9]. Second, the high flow washes the nasopharyngeal dead space, thus reducing CO_2_ re-breathing; this effect reduces respiratory rate and minute ventilation [10]. Third, the small amount of PEEP may reduce lung collapse [11], enabling better gas exchange and reduced work of breathing; moreover, in patients with chronic obstructive pulmonary disease (COPD), this level of PEEP may counterbalance autoPEEP, further reducing the work of breathing [12–14]. Finally, humidification may improve mucus drainage and reduce mucus retention, alleviating the associated atelectasis [15, 16].

HFNC after extubation has shown benefits in patients at low risk for extubation failure [17], in mixed populations of critically ill patients [9, 18], and in patients after cardiothoracic surgery [19], but not in post-cardiac obese patients [20]. We hypothesized that HFNC as compared to standard oxygen may reduce postextubation respiratory failure in non-hypercapnic patients at high risk for extubation failure. We focused the current study on patients with high risk for extubation failure excluding hypercapnic patients in whom the use of noninvasive ventilation (NIV) may be beneficial [21, 22].

## Methods

This randomized trial (Clinicaltrials.gov NCT01820507) was conducted in four general ICUs in Spain in 2013–2014. Approval for involvement of human subjects was obtained from institutional review boards (IRBs) at each study sites [FA IRB No. CEIC 12/85, HG IRB No. A06-13, HSLl IRB No. 2105/13, HVH IRB No. PR(AG)116/2013]. Written informed consent was obtained from patients’ relatives.

We screened adult patients receiving MV >12 h deemed ready for scheduled extubation after a spontaneous breathing trial (SBT). We included patients fulfilling at least one of the following high-risk criteria for extubation failure [20–24]: older than 65 years, heart failure as cause of intubation, non-hypercapnic moderate-to-severe COPD, APACHE II score >12 points at extubation, body mass index >30 kg/m^2^, weak cough and copious secretions, more than one SBT failure, or MV >7 days. We excluded patients with tracheotomy, inability to follow commands, or do-not-reintubate orders, as well as those who developed hypercapnia during the SBT because they required NIV immediately after extubation.

### Weaning protocol

Patients fulfilling the criteria for tolerance of spontaneous ventilation underwent an SBT following the local protocols. The SBT ranged from 30 to 120 min and was performed with 5-cmH_2_O continuous positive airway pressure, 7-cmH_2_O pressure support, or T-tube.

Criteria for SBT failure were agitation, anxiety, depressed mental status, diaphoresis, cyanosis, evidence of increasing respiratory effort, increased accessory muscle activity, facial signs of distress, dyspnea, PaO_2_ ≤60 mmHg or SpO_2_ <90% on FiO_2_ ≥0.5, PaCO_2_ >50 mmHg or >8 mmHg increase, arterial pH <7.32 or ≥0.07 decrease, respiratory rate >35 breaths min^−1^ or ≥50% increase, heart rate >140 beats min^−1^ or ≥20% increase, systolic arterial pressure >180 mmHg or ≥20% increase, systolic arterial pressure <90 mmHg, or cardiac arrhythmia.

Patients who failed the SBT were reconnected to the ventilator for an additional 24-h rest period before a new SBT. Patients who tolerated the SBT were directly extubated and randomized to receive either high-flow or conventional oxygen therapy for a fixed 24-h period. Randomization was performed via a computerized random-number table in blocks of four for each hospital; allocation was concealed through numbered opaque envelopes.

### Interventions

#### Conventional group

Oxygen after extubation was supplied either by nasal prongs or facial mask with oxygen concentration regulated by Venturi effect.

#### HFNC group

Oxygen after extubation was supplied by Optiflow^®^ (Fisher&Paykel, New Zealand). Flow was started at 40 L/min and was adjusted according to patients’ subjective tolerance. The humidifier was set in the invasive mode (37 °C), but was switched to noninvasive mode (34 °C) if the patient felt excessive warmth.

In both groups, oxygen supply was continuously adjusted to achieve SpO2 between 92 and 95%. At the end of the 24-h study period, all patients received conventional oxygen therapy when needed and were followed up to hospital discharge.

The primary outcome variable was respiratory failure within 72 h postextubation, defined as the presence and persistence of any of the following: respiratory acidosis (pH <7.35 with PaCO_2_ >45 mmHg), hypoxemia (SpO_2_ <90% or PaO_2_ <60 mmHg with FiO_2_ ≥0.5), tachypnea >35 breaths/min and/or signs of respiratory muscle fatigue, and/or low level of consciousness or agitation.

Patients were continuously monitored by electrocardiography and pulse oximetry. For the purpose of this trial, NIV as rescue treatment for extubation failure was discouraged, but remained available at the discretion of the attending team.

Secondary outcome variables were reintubation, ICU and hospital lengths of stay, and survival. Criteria for immediate reintubation were cardiac or respiratory arrest, respiratory pauses with neurological deterioration, massive aspiration, uncontrollable agitation, sputum retention, and hemodynamic deterioration unresponsive to vasoactive drugs. Patients were also reintubated when they needed it for non-respiratory reasons, such as emergency surgery or when postextubation respiratory failure did not improve after 12 h.

### Statistical analysis

With an expected extubation failure rate of 28% in the control group and an absolute expected improvement with HFNC of 7% (25% relative reduction) [25], the planned sample was 592 patients in each arm, for an alpha error of 5% and a power of 80%.

Because it was impossible to mask patients and staff to treatment and outcome, we used the following measures to minimize bias in assessing results: The database was monitored by third parties with no direct involvement in the study procedures and no interest in outcome, and the data were analyzed exactly according to the statistical analysis plan decided on before the study started.

Data were analyzed with an intention-to-treat approach. Categorical variables were compared by Chi-square or Fisher’s exact test, as appropriate. Continuous variables were compared by Student’s *t* test. Kaplan–Meier survival analyses were done for extubation failure, reintubation, and mortality, and the log-rank test was used for comparisons.

To determine factors independently associated with postextubation respiratory failure, we elaborated a multivariable logistic regression model using a backward procedure, including HFNC and all non-redundant variables associated with postextubation respiratory failure (*p* < 0.1). Statistics were analyzed with STATA 10.0^®^ (StataCorp, TX) and EpiInfo-7^®^ (CDC, GA).

## Results

**Fig. 1:**
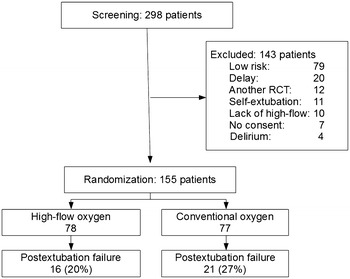
CONSORT flowchart of the study

When after 18 months only 155 patients had been recruited (78 randomized to receive high-flow oxygen and 77 conventional oxygen), the investigators stopped the trial due to low recruitment (Fig. 1).

The two groups were not different at inclusion (Table 1). The most common criteria for high-risk for postextubation respiratory failure were age >65 years and abundant secretions. All patients tolerated 24-h HFNC, but 14 (18%) reported some kind of discomfort, mainly noise, and 2 (2.6%) developed small nostril skin lesions. Pneumonia after extubation was the only reported adverse event, affecting only 2 (2.6%) patients, both in the conventional group.

**Table 1:** Characteristics at randomization of patients in the HFNC group versus those in the conventional oxygen therapy group

Baseline variables	HFNC *n* = 78	Conventional *n* = 77	*p*
Age (years)	67.3 ± 12.1	69.7 ± 13.0	0.2
Female sex	32 (41%)	22 (29%)	0.1
Height (cm)	168 ± 9	168 ± 21	0.2
APACHE II on admission, points	21 ± 8.8	21 ± 8.2	0.9
APACHE II at extubation, points	11 ± 5.5	10 ± 6.7	0.2
Length of mechanical ventilation before extubation (days)	8.2 ± 5.9	7.4 ± 3.6	0.3
High-risk criteria^a^			
Age >65 years	49 (67%)	55 (75%)	0.4
Abundant secretions	33 (47%)	35 (51%)	0.7
>2 comorbidities	31 (43%)	34 (49%)	0.5
APACHE II >12 points	24 (34%)	31 (45%)	0.2
Body mass index >30 kg/m^2^	14 (20%)	18 (25%)	0.5
Chronic obstructive pulmonary disease	12 (18%)	10 (15%)	0.8
Weak cough	10 (15%)	14 (21%)	0.4
Congestive heart failure	9 (14%)	9 (14%)	1
Failed spontaneous breathing trial	6 (9%)	5 (7%)	1
Pre-SBT respiratory rate (min^−1^)	21.7 ± 6.0	21.8 ± 5.8	0.8
Pre-SBT FiO_2_	0.30 ± 0.08	0.30 ± 0.08	0.9
Pre-SBT SpO_2_ (%)	96.5 ± 1.9	96.0 ± 2.3	0.8

*HFNC* high-flow nasal cannula, *APACHE II* Acute Physiology and Chronic Health Evaluation, *SBT* spontaneous breathing trial

^a^More than one criteria can be present

**Fig. 2:**
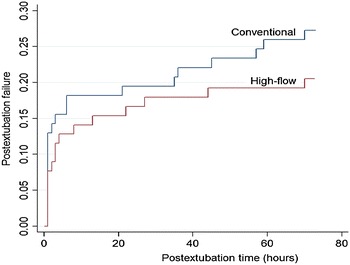
Kaplan–Meier plot of 72-h postextubation failure in patients receiving HFNC versus conventional oxygen therapy. The difference did not reach statistical significance (*p* = 0.2)

Postextubation respiratory failure developed in 16 (20%) HFNC patients and in 21 (27%) conventional patients [OR 0.69 (95% CI 0.31–1.54), *p* = 0.2] (Table 2; Fig. 2). Time-to-failure was not different in the two groups [17 (7, 44) h vs. 21 (6, 44) h, *p* = 0.7]. The criteria identifying respiratory failure were not different between groups. NIV was used as rescue therapy for respiratory failure in 10 (62%) HFNC patients and 12 (57%) conventional patients (*p* = 0.9).

**Table 2:** Outcome variables in the two groups

Outcome variables	HFNC *n* = 78	Conventional *n* = 77	*p*
Postextubation respiratory failure	16 (20%)	21 (27%)	0.2
Causes of respiratory failure			
Hypoxemia	11 (65%)	14 (67%)	0.6
Respiratory rate >35	9 (54%)	14 (67%)	0.3
Respiratory muscle fatigue	7 (47%)	8 (53%)	0.5
Respiratory acidosis	2 (12%)	8 (36%)	0.08
Low level of consciousness	3 (18%)	1 (5%)	0.2
Time-to-failure (h)	17 [7, 44]	21 [6, 44]	0.7
Reintubation within 72 h	9 (11%)	12 (16%)	0.5
Intensive care unit length of stay (days)	12 [7, 25]	14 [9, 17]	0.8
Intensive care unit mortality	6 (7.7%)	7 (9.0%)	0.9
Hospital length of stay (days)	27 [18, 54]	27 [18, 47]	1
Hospital mortality	12 (15.4%)	12 (15.6%)	1

Reintubation was needed in 9 (11%) HFNC patients and in 12 (16%) conventional patients [OR 0.71 (95% CI 0.25–1.95), *p* = 0.5]. Reintubation was needed in 3 (30%) HFNC patients treated with NIV and in 7 (58%) conventional patients treated with NIV (*p* = 0.18).

Length of ICU and hospital stays and mortality were not different between the two groups. Mortality in patients exhibiting postextubation respiratory failure did not differ between those treated with NIV and those without (10/22, 45% and 3/15, 20%; *p* > 0.1, respectively).

The multivariable logistic regression model identified HFNC [OR 0.43 (0.18–0.99), *p* = 0.04) and cancer [OR 2.87 (1.04–7.91), *p* = 0.04] as independently associated with postextubation respiratory failure (see Additional file 1). Due to the limited sample size, the multivariable logistic regression must be considered as exploratory. In order to explore the likelihood of a clinically sound effect of HFNC, a sensitivity analysis with four different regression models is shown in the Additional file 1.

## Discussion

Given our small sample, postextubation respiratory failure with HFNC was not significantly different than with conventional oxygen. Nevertheless, after adjustment for confounding variables in four multivariable regression models, HFNC might be independently associated with lower postextubation failure.

Extubation failure remains one of the most pressing issues in MV. Despite advances in protective ventilation, sedation practices, and early mobilization, 10–20% of patients experience extubation failure [3]. Moreover, extubation failure is clearly associated with increased morbidity and mortality. Mortality rate may indeed reach 50% in patients that require reintubation [24]. The incidence of postextubation respiratory failure is clearly dependent on ICU case-mix, being lower in patients intubated for scheduled surgery and higher in medical and debilitated patients. Therefore, it is essential to classify patients according to risk when testing any preventive treatment. There is no general consensus about the risk factors that predict extubation failure [1, 2, 23], and different investigators have defined their own criteria. Recently, Thille et al. [24] demonstrated that caregivers’ experience is of limited value in predicting extubation failure; only one-third of the patients who required reintubation were considered at high risk for extubation failure in a very experienced ICU. In our study, we used nine criteria to select patients with higher likelihood of failure. Our 27% postextubation respiratory failure rate in the conventional group is very close to our anticipated rate and suggests that less sick patients were excluded.

Although supportive treatments may help prevent respiratory failure, they may also delay intubation in patients who develop respiratory failure. Esteban et al. [26] found increased mortality rate in patients receiving NIV to treat postextubation respiratory failure and attributed this finding mainly to a delay in reintubation. In a different scenario, Kang et al. [27] reported that patients intubated after 120 h of HFNC had a higher mortality than those intubated before 48 h, thereby suggesting that prolonging HFNC unduly is clearly detrimental to the patients [28]. However, these studies focused on supportive treatment used to treat respiratory failure rather than to prevent it. Although supportive treatment might mask signs and symptoms of respiratory failure that might delay intubation, our data showed no delays in reintubation in high-flow patients.

Our study might also shed light on the role of NIV as rescue treatment for postextubation respiratory failure. The literature supports NIV in hypercapnic patients [22], and this was the rationale for excluding them in our trial. By contrast, studies on the use of NIV as rescue treatment for patients without hypercapnia have found discrepant results; thus, we discouraged its use, but allowed it at the discretion of the attending team. Nevertheless, nearly half the patients who developed postextubation respiratory failure received NIV. There was a trend toward a lower reintubation rate in patients rescued with NIV, but there was also a trend toward higher mortality. One can speculate that physicians commonly offered NIV to the sickest patients, precluding any conclusion about the beneficial or harmful effect of NIV in this setting.

### Limitations of the study

The small sample size is the major limitation of our study. Enrollment was much lower than expected for various reasons. First, after their initial commitment to participate, some centers decided to opt out due to workforce reductions. Second, number of devices were insufficient at some centers. Third, budget constraints at some centers resulted in a shortage of circuits and disposables. Nevertheless, there is growing evidence that robust statistical analyses with covariate adjustment in multiple regression models can help reduce sample size requirements [29]. All our exploratory regression models are prone to “overfitting” due to sample size, but they may offer some insights about the likelihood of a real effect of high flow. Additionally, very recently Hernandez et al. [17] demonstrated similar results in low-risk patients in a study with an adequate sample size.

The optimal length of HFNC treatment after extubation is not yet known. We decided upon a 24-h interval for practical reasons. Others have used HFNC for 48-h [9]. Further studies are required to determine the adequate length of HFNC duration after extubation. Availability of the device in the ICUs may be a limiting factor for a prolonged used of HFNC in this indication.

Our use of conventional oxygen in the control arm also deserves comment. Some studies suggest that high-risk patients have better outcome after extubation if routinely treated with NIV [10, 30], but others suggest otherwise [26, 31]. This issue remains controversial, and NIV has a definite indication only in hypercapnic patients [22, 32]. Thus, we excluded hypercapnic patients from our study. Some ongoing trials comparing HFNC with NIV in postextubation failure may help define the best comparison arm.

Our lack of sequential recordings of arterial blood gases and respiratory variables precludes any speculation about the physiological features involved in the improvement in respiratory failure, but published studies show HFNC improves oxygenation and thoracoabdominal synchrony and decreases respiratory rate and dyspnea [8–11, 33, 34].

## Conclusion

Although exploratory multivariable logistic regression analysis found a protective effect of HFNC against postextubation respiratory failure, our study is inconclusive as to a potential benefit of HFNC over conventional oxygen to prevent occurrence of respiratory failure in non-hypercapnic patients at high risk for extubation failure.

## Additional file

**Additional file 1.** Digital Content.

## Abbreviations

COPD: chronic obstructive pulmonary disease
HFNC: high-flow nasal cannula
ICU: intensive care unit
MV: mechanical ventilation
NIV: noninvasive ventilation
PEEP: positive end-expiratory pressure
SBT: spontaneous breathing trial
