# Supplementary material for: High-flow nasal cannula to prevent postextubation respiratory failure in high-risk non-hypercapnic patients: a randomized multicenter trial
Source: Ann Intensive Care. 2017 May 2;7:47. doi: 10.1186/s13613-017-0270-9 (PMC5413462; doi:10.1186/s13613-017-0270-9)
Supplement: Supplementary file 1 — Additional file 1. Digital Content. [file 13613_2017_270_MOESM1_ESM.doc]

**High-flow oxygen to prevent postextubation respiratory failure in high-risk patients: a randomized multicenter trial**

SUPPLEMENTAL DIGITAL CONTENT

RESULTS:

We found 4 different multivariable regression models able to detect High-flow therapy as a variable independently associated with lower postextubation failure.

Despite the possibility of overfitting, the 4 models showed nonsignificant Hosmer-Lemeshow goodness-of-fit tests, suggesting good calibration.

**Model 01.**

**Logistic regression for Postextubation Failure with 6 variables: High-flow, Cancer, DaysPreviousMV, PreCOPD, BMI, and HeartFailure.**

Logistic regression Number of obs = 142

LR chi2(6) = 8.12

Prob > chi2 = 0.2295

Log likelihood = -72.926 Pseudo R2 = 0.0527

--------------------------------------------------------------------------------

Failure | Odds Ratio Std. Err. z P>|z| [95% Conf. Interval]

---------------+----------------------------------------------------------------

1.High-flow| .43 .1847 -1.96 0.049 .184 .997

1.Cancer | 2.87 1.4852 2.04 0.041 1.044 7.915

DaysPreviousMV| 1.02 .0400 0.64 0.523 .949 1.106

1.PreCOPD | 1.77 .8953 1.14 0.255 .661 4.770

1.BMI | 1.06 .5127 0.12 0.902 .411 2.735

1.HeartFailure | 1.26 .6880 0.44 0.663 .437 3.673

Constant | .24 .1119 -3.07 0.002 .098 .599

--------------------------------------------------------------------------------

. estat gof, all group (10) table

Logistic model for Failure, goodness-of-fit test

(Table collapsed on quantiles of estimated probabilities)

+--------------------------------------------------------+

| Group | Prob | Obs_1 | Exp_1 | Obs_0 | Exp_0 | Total |

|-------+--------+-------+-------+-------+-------+-------|

| 1 | 0.1120 | 2 | 1.6 | 13 | 13.4 | 15 |

| 2 | 0.1204 | 1 | 1.6 | 13 | 12.4 | 14 |

| 3 | 0.1649 | 2 | 1.9 | 12 | 12.1 | 14 |

| 4 | 0.2112 | 3 | 2.9 | 12 | 12.1 | 15 |

| 5 | 0.2327 | 4 | 2.9 | 9 | 10.1 | 13 |

|-------+--------+-------+-------+-------+-------+-------|

| 6 | 0.2455 | 7 | 3.6 | 8 | 11.4 | 15 |

| 7 | 0.2659 | 1 | 3.6 | 13 | 10.4 | 14 |

| 8 | 0.3053 | 2 | 4.0 | 12 | 10.0 | 14 |

| 9 | 0.3688 | 3 | 4.6 | 11 | 9.4 | 14 |

| 10 | 0.5173 | 8 | 6.3 | 6 | 7.7 | 14 |

+--------------------------------------------------------+

number of observations = 142

number of groups = 10

Hosmer-Lemeshow chi2(8) = 10.86

Prob > chi2 = 0.2100

**Model 02**

**Logistic regression for Postextubation Failure with 6 variables: High-flow, Cancer, DaysPreviousMV, PreCOPD, BMI, and HeartFailure, and one interaction (BMI and DaysPreviousMV).**

Logistic regression Number of obs = 142

LR chi2(7) = 8.13

Prob > chi2 = 0.3216

Log likelihood = -72.922 Pseudo R2 = 0.0528

--------------------------------------------------------------------------------------

Failure | Odds Ratio Std. Err. z P>|z| [95% Conf. Interval]

---------------------+----------------------------------------------------------------

1.High-flow | .43 .1849 -1.96 0.050 .185 .999

1.Cancer | 2.89 1.4849 2.04 0.042 1.040 7.912

DaysPreviousMV | 1.02 .0429 0.57 0.572 .943 1.112

1.PreCOPD | 1.77 .8945 1.14 0.256 .660 4.766

1.BMI | .98 1.0554 -0.02 0.986 .119 8.075

1.HeartFailure | 1.27 .6876 0.43 0.664 .436 3.671

BMI#c.DaysPreviousMV |

1 | 1.01 .1162 0.08 0.935 .805 1.265

Constant | .25 .1175 -2.94 0.003 .096 .628

------------------------------------------------------------------------------------

. estat gof, all group (10) table

Logistic model for Failure, goodness-of-fit test

(Table collapsed on quantiles of estimated probabilities)

+--------------------------------------------------------+

| Group | Prob | Obs_1 | Exp_1 | Obs_0 | Exp_0 | Total |

|-------+--------+-------+-------+-------+-------+-------|

| 1 | 0.1122 | 2 | 1.6 | 13 | 13.4 | 15 |

| 2 | 0.1202 | 1 | 1.6 | 13 | 12.4 | 14 |

| 3 | 0.1659 | 2 | 1.9 | 12 | 12.1 | 14 |

| 4 | 0.2115 | 2 | 2.7 | 12 | 11.3 | 14 |

| 5 | 0.2306 | 5 | 3.1 | 9 | 10.9 | 14 |

|-------+--------+-------+-------+-------+-------+-------|

| 6 | 0.2442 | 7 | 3.6 | 8 | 11.4 | 15 |

| 7 | 0.2667 | 1 | 3.6 | 13 | 10.4 | 14 |

| 8 | 0.3048 | 3 | 4.0 | 11 | 10.0 | 14 |

| 9 | 0.3696 | 2 | 4.6 | 12 | 9.4 | 14 |

| 10 | 0.5297 | 8 | 6.3 | 6 | 7.7 | 14 |

+--------------------------------------------------------+

number of observations = 142

number of groups = 10

Hosmer-Lemeshow chi2(8) = 12.40

Prob > chi2 = 0.1341

**Model 03**

**Logistic regression for Postextubation Failure with 5 variables: High-flow, Cancer, DaysPreviousMV, PreCOPD, and BMI, and one interaction (Cancer and DaysPreviousMV).**

Logistic regression Number of obs = 142

LR chi2(6) = 11.55

Prob > chi2 = 0.0728

Log likelihood = -71.2109 Pseudo R2 = 0.0750

-----------------------------------------------------------------------------------------

Failure | Odds Ratio Std. Err. z P>|z| [95% Conf. Interval]

------------------------+----------------------------------------------------------------

1.High-flow | .41 .1794 -2.04 0.042 .173 .966

1.Cancer | .40 .4921 -0.74 0.457 .037 4.397

DaysPreviousMV | .99 .0466 -0.10 0.917 .908 1.091

1.PreCOPD | 1.86 .9505 1.23 0.220 .688 5.064

1.BMI | 1.01 .5031 0.03 0.978 .383 2.681

Cancer#c.DaysPreviousMV |

1 | 1.26 .1635 1.80 0.071 .980 1.628

Constant | .33 .1607 -2.28 0.023 .127 .857

-----------------------------------------------------------------------------------------

. estat gof, all group (10) table

Logistic model for Failure, goodness-of-fit test

(Table collapsed on quantiles of estimated probabilities)

+--------------------------------------------------------+

| Group | Prob | Obs_1 | Exp_1 | Obs_0 | Exp_0 | Total |

|-------+--------+-------+-------+-------+-------+-------|

| 1 | 0.1141 | 2 | 1.7 | 13 | 13.3 | 15 |

| 2 | 0.1165 | 1 | 1.6 | 13 | 12.4 | 14 |

| 3 | 0.1185 | 2 | 1.6 | 12 | 12.4 | 14 |

| 4 | 0.1960 | 5 | 2.5 | 9 | 11.5 | 14 |

| 5 | 0.2372 | 1 | 3.0 | 13 | 11.0 | 14 |

|-------+--------+-------+-------+-------+-------+-------|

| 6 | 0.2410 | 1 | 3.6 | 14 | 11.4 | 15 |

| 7 | 0.2440 | 6 | 3.4 | 8 | 10.6 | 14 |

| 8 | 0.2481 | 4 | 3.4 | 10 | 10.6 | 14 |

| 9 | 0.3770 | 4 | 4.9 | 10 | 9.1 | 14 |

| 10 | 0.7946 | 7 | 7.3 | 7 | 6.7 | 14 |

+--------------------------------------------------------+

number of observations = 142

number of groups = 10

Hosmer-Lemeshow chi2(8) = 10.46

Prob > chi2 = 0.2341

**Model 04**

**Logistic regression for Postextubation Failure with 5 variables: High-flow, Cancer, DaysPreviousMV, PreCOPD, and BMI, and 2 interactions (Cancer and DaysPreviousMV and BMI and PreCOPD).**

Logistic regression Number of obs = 142

LR chi2(7) = 11.99

Prob > chi2 = 0.1008

Log likelihood = -70.9896 Pseudo R2 = 0.0779

-----------------------------------------------------------------------------------------

Failure | Odds Ratio Std. Err. z P>|z| [95% Conf. Interval]

------------------------+----------------------------------------------------------------

1.High-flow | .41 .1824 -2.00 0.045 .175 .982

1.Cancer | .41 .4984 -0.73 0.464 .038 4.410

DaysPreviousMV | .99 .0459 -0.09 0.928 .910 1.090

1.PreCOPD | 2.18 1.2141 1.40 0.161 .732 6.494

1.BMI | 1.20 .6583 0.33 0.744 .407 3.517

Cancer#c.DaysPreviousMV |

1 | 1.26 .1623 1.79 0.073 .978 1.621

BMI#PreCOPD |

1 1 | .43 .5665 -0.64 0.522 .032 5.720

Constant | .31 .1541 -2.36 0.018 .119 .8218

-----------------------------------------------------------------------------------------

. estat gof, all group (10) table

Logistic model for Failure, goodness-of-fit test

(Table collapsed on quantiles of estimated probabilities)

+--------------------------------------------------------+

| Group | Prob | Obs_1 | Exp_1 | Obs_0 | Exp_0 | Total |

|-------+--------+-------+-------+-------+-------+-------|

| 1 | 0.1110 | 2 | 1.6 | 13 | 13.4 | 15 |

| 2 | 0.1131 | 1 | 1.6 | 13 | 12.4 | 14 |

| 3 | 0.1301 | 2 | 1.7 | 12 | 12.3 | 14 |

| 4 | 0.2145 | 3 | 2.4 | 11 | 11.6 | 14 |

| 5 | 0.2296 | 3 | 3.1 | 11 | 10.9 | 14 |

|-------+--------+-------+-------+-------+-------+-------|

| 6 | 0.2337 | 3 | 3.7 | 13 | 12.3 | 16 |

| 7 | 0.2367 | 2 | 3.1 | 11 | 9.9 | 13 |

| 8 | 0.2675 | 8 | 3.7 | 6 | 10.3 | 14 |

| 9 | 0.4014 | 3 | 4.8 | 11 | 9.2 | 14 |

| 10 | 0.8112 | 6 | 7.3 | 8 | 6.7 | 14 |

+--------------------------------------------------------+

number of observations = 142

number of groups = 10

Hosmer-Lemeshow chi2(8) = 9.61

Prob > chi2 = 0.2938
